# Controversial Nomenclature in Peptide Synthesis: A Call for Clarity

**DOI:** 10.1002/psc.70044

**Published:** 2025-07-27

**Authors:** Anamika Sharma, Ashish Kumar, Beatriz G. de la Torre, Fernando Albericio

**Affiliations:** ^1^ Peptide Science Laboratory, School of Chemistry and Physics University of KwaZulu‐Natal Durban South Africa; ^2^ KwaZulu‐Natal Research Innovation and Sequencing Platform (KRISP), School of Laboratory Medicine and Medical Sciences, College of Health Sciences University of KwaZulu‐Natal Durban South Africa; ^3^ Department of Organic Chemistry University of Barcelona Barcelona Spain

## Abstract

Various approaches to make peptides have been adopted globally owing to their high demand. The three main approaches commonly used for this purpose are solution synthesis, also called classical solution‐phase peptide synthesis (CSPS), solid‐phase peptide synthesis (SPPS), and liquid‐phase peptide synthesis (LPPS). Each method offers unique advantages: CSPS for scalability, SPPS for automation and efficiency, and LPPS for combining solution‐phase simplicity with iterative synthesis using soluble tags.

## Graphical abstract

The three main approaches commonly employed for peptide synthesis are solution synthesis or also called classical solution‐phase peptide synthesis (CSPS), solid‐phase peptide synthesis (SPPS), and liquid‐phase peptide synthesis (LPPS).

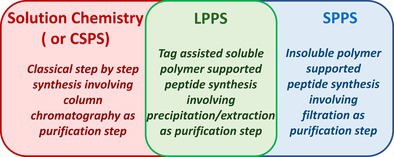

Peptides have gained substantial attention as therapeutic agents because of their high target selectivity, excellent efficacy, and relatively low toxicity [1, 2, 3]. They bridge the gap between small molecules and biologics and may offer better characteristics than both [2, 4]. Peptide‐based drugs are being successfully developed and have been approved for several targets like metabolic disorders, infectious diseases, cancer, and autoimmune conditions [5]. With around 100 peptide drugs on the market and more than 600 in clinical trials, the medical and commercial relevance of these molecules is significant [6, 7]. The peptide market is expanding exponentially, demanding effective approaches and strategies to prepare on an industrial scale [8]. In this regard, strategies for peptide synthesis have evolved. According to the literature, early peptide synthesis methods relied entirely on solution‐phase chemistry that was performed in solution, in which two monomers are reacted to obtain material from which the product is isolated via various separation techniques like column chromatography [9]. No terminology was given for such protocols which resembled chemistry that was used to synthesize small molecules. Solution chemistry (or synthesis) thus marked the *first wave of peptide synthesis*. This strategy is scalable but often more tedious and time‐consuming due to the isolation and purification steps involved in each step [9, 10]. To address this issue, in 1963, Merrifield introduced the concept of an insoluble polymeric protecting group to carry out peptide synthesis on a solid support [10, 11]. As the reaction was carried out in heterogeneous reaction media, simple filtration was adopted as the purification step to eliminate the unreacted reagents and soluble by‐products. This approach bypassed the tedious and time‐consuming purification steps, thereby leading to significant time savings. This strategy allows the use of excess of reagents to drive reactions to completion. This approach therefore was referred to as *solid‐phase peptide synthesis (SPPS)*. This strategy marked the *second wave of peptide synthesis*, owing to the expediency of the process, allowing the preparation of a given peptide in only a few hours. SPPS can be scaled to multikilogram production on the industrial level and automated peptide synthesizers [8]. A major issue with the SPPS is the use of excess reagents and large amounts of solvents for the resin washing process. As a result, it generates significant solvent waste and does not align with the 12 green principles [12, 13]. Recent years have witnessed a spike in life cycle assessment (LCA), which is related to the synthetic processes for peptide synthesis on a day‐to‐day basis [13]. The SPPS strategies can be expensive and lack sustainability. Due to the ease of synthesis, SPPS still remains the choice for peptide synthesis at research and large scale.

Another approach was adopted employing hydrophobic or hydrophilic but soluble protecting groups enabling reactions to be performed in solution [14, 15]. This approach of tag‐assisted peptide synthesis was referred to as *liquid‐phase peptide synthesis (LPPS)* and marks the onset of the *third wave of peptide synthesis* [16]. This approach offers advantages over both solution chemistry and SPPS [15]. In this regard, LPPS reactions are performed in solution at stoichiometric ratios of reactants, and purification of the intermediates is achieved using either precipitation or extraction. Like SPPS, LPPS eliminates tedious and time‐consuming column purification. The LPPS approach was first introduced by Bayer and Mutter in 1972 using polyethylene glycols (PEGs) as the soluble support or tag (Figure 1). In their seminal paper, Bayer and Mutter coined the term LPPS [15].

**FIGURE 1:**
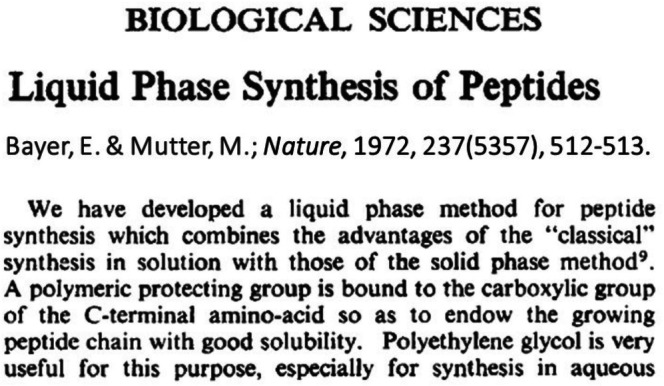
Snippet of the early publication of Bayer and Mutter in 1972 on LPPS [15].

The LPPS strategy resembles that of solution chemistry. Inspired by SPPS, LPPS employs a soluble support to enable purification at each step by precipitation/extraction (unlike the need for filtration in the case of SPPS) [16]. In LPPS, protected amino acids are added iteratively (similar to SPPS). The LPPS technique went unused for several decades since its inception in the 1970s. Owing to lower solvent consumption and waste generation (as calculated by process mass intensity [PMI] and complete environmental factor [cEF]) relative to SPPS, LPPS has gained popularity with the development of novel tags over the past few years [13].

The terms “solution chemistry” and “LPPS” are often inaccurately used interchangeably in the literature [17, 18]. With the advent of LPPS, our group referred to solution chemistry as *classical solution peptide synthesis (CSPS)* [16] while others used the term solution‐phase peptide synthesis (SolPPS) [17]. The term “classical” was employed in the earlier publication of Bayer and Mutter, who sought to differentiate LPPS involving PEGs as tags from solution chemistry (Figure 1) [14, 15].

In this context, we advocate for referring to solution synthesis as simply solution synthesis or CSPS. The term LPPS should be reserved for the tag‐assisted strategy, which, although performed in solution, aligns more closely with the philosophy of SPPS (Figure 2). Following this terminology, hybrid approaches—exemplified by the synthesis of Tirzepatide by Eli Lilly, in which protected fragments are prepared by SPPS and subsequently assembled in solution—may be termed “hybrid SPPS‐solution” or “hybrid SPPS‐CSPS” [19]. Similarly, if the fragments were synthesized using LPPS techniques, the process should be referred to as “hybrid LPPS‐solution” or “hybrid LPPS‐CSPS.”

**FIGURE 2:**
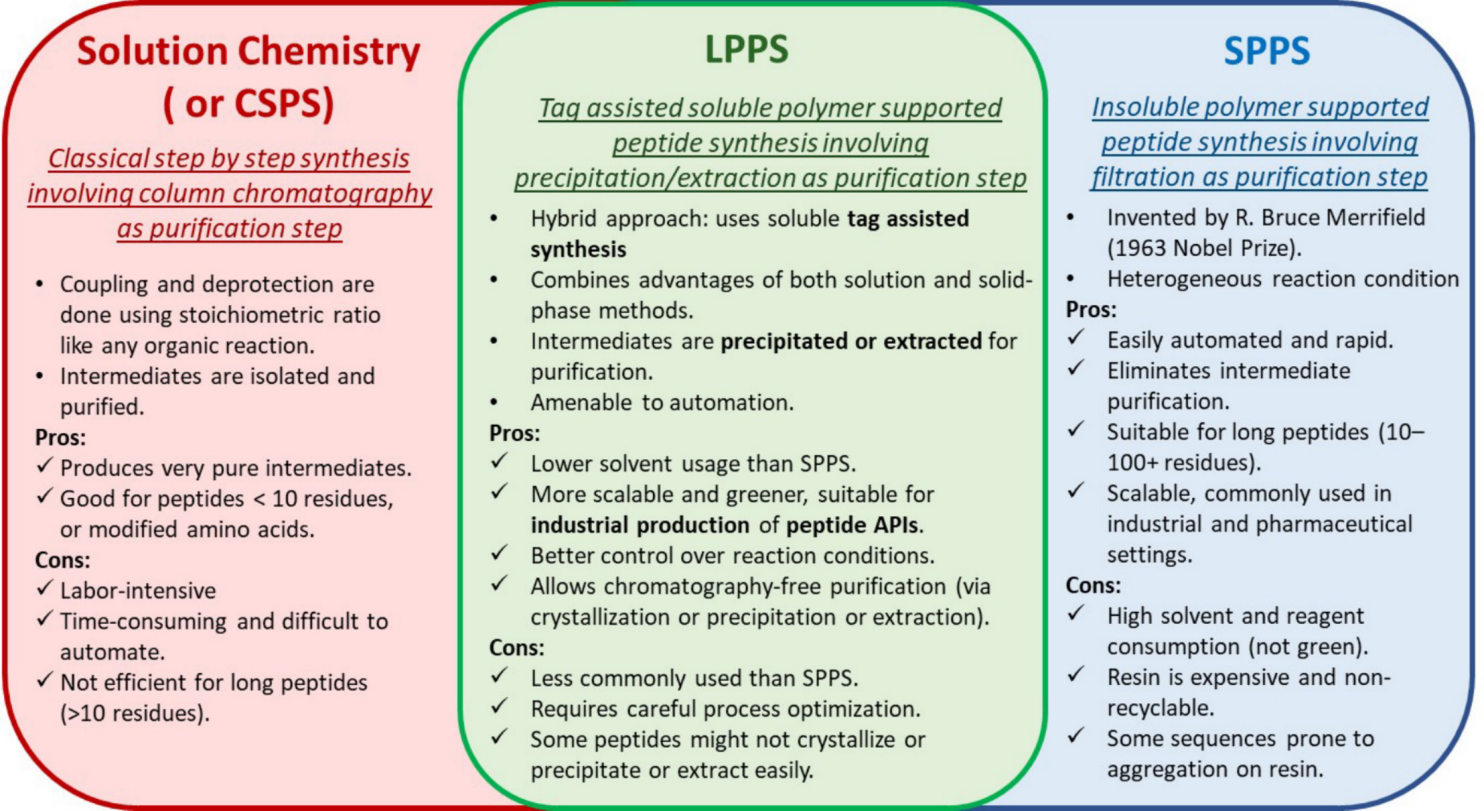
Salient features and differences explaining the nomenclature between the three approaches used for peptide synthesis.

## Conflicts of Interest

The authors declare no conflicts of interest.

## Data Availability

There are no data associated with this manuscript, but all the data generated by the research group are always available upon request.
